# Overview of the Spinal Cord Injury – Quality of Life (SCI-QOL) measurement system

**DOI:** 10.1179/2045772315Y.0000000023

**Published:** 2015-05

**Authors:** David S. Tulsky, Pamela A. Kisala, David Victorson, Denise G. Tate, Allen W. Heinemann, Susan Charlifue, Steve C. Kirshblum, Denise Fyffe, Richard Gershon, Ann M. Spungen, Charles H. Bombardier, Trevor A. Dyson-Hudson, Dagmar Amtmann, Claire Z. Kalpakjian, Seung W. Choi, Alan M. Jette, Martin Forchheimer, David Cella

**Affiliations:** 1 Department of Physical Therapy, University of Delaware, College of Health Sciences, Newark, DE, USA; 2 Kessler Foundation, West Orange, NJ, USA; 3 Northwestern University Feinberg School of Medicine, Chicago, IL, USA; 4 University of Michigan Medical School, Ann Arbor, MI, USA; 5 Rehabilitation Institute of Chicago, Chicago, IL, USA; 6 Craig Hospital, Englewood, CO, USA; 7 Kessler Institute for Rehabilitation, West Orange, NJ, USA; 8 New Jersey Medical School, Rutgers University, Newark, NJ, USA; 9 James J. Peters VA Medical Center, Bronx, NY, USA; 10 University of Washington, Seattle, WA, USA; 11 CTB-McGraw Hill, LLC, Monterey, CA, USA; 12 Boston University School of Public Health, Boston, MA, USA

**Keywords:** Computer Adaptive Testing, Health-Related Quality of Life, Item Response Theory, Patient Reported Outcomes, Spinal Cord Injury

## Abstract

**Context/Objective:**

The Spinal Cord Injury – Quality of Life (SCI-QOL) measurement system was developed to address the shortage of relevant and psychometrically sound patient reported outcome (PRO) measures available for clinical care and research in spinal cord injury (SCI) rehabilitation. Using a computer adaptive testing (CAT) approach, the SCI-QOL builds on the Patient Reported Outcomes Measurement Information System (PROMIS) and the Quality of Life in Neurological Disorders (Neuro-QOL) initiative. This initial manuscript introduces the background and development of the SCI-QOL measurement system. Greater detail is presented in the additional manuscripts of this special issue.

**Design:**

Classical and contemporary test development methodologies were employed. Qualitative input was obtained from individuals with SCI and clinicians through interviews, focus groups, and cognitive debriefing. Item pools were field tested in a multi-site sample (*n* = 877) and calibrated using item response theory methods. Initial reliability and validity testing was performed in a new sample of individuals with traumatic SCI (*n* = 245).

**Setting:**

Five Model SCI System centers and one Department of Veterans Affairs Medical Center across the United States.

**Participants:**

Adults with traumatic SCI.

**Interventions:**

n/a

**Outcome Measures:**

n/a

**Results:**

The SCI-QOL consists of 19 item banks, including the SCI-Functional Index banks, and 3 fixed-length scales measuring physical, emotional, and social aspects of health-related QOL (HRQOL).

**Conclusion:**

The SCI-QOL measurement system consists of psychometrically sound measures for individuals with SCI. The manuscripts in this special issue provide evidence of the reliability and initial validity of this measurement system. The SCI-QOL also links to other measures designed for a general medical population.

## Introduction

Traumatic spinal cord injury (SCI) is acute, unexpected, and dramatically alters the course of an individual's life. It causes sudden, often devastating damage to the central nervous system, with potential adverse effects in multiple body systems including musculoskeletal, integumentary, digestive, urinary, cardiovascular, and reproductive. Many of the secondary complications experienced by individuals with SCI are quite unlike those experienced by persons with general health issues or other neurological disorders.^1^ People with SCI must relearn basic skills such as eating, bathing, dressing, and driving. Living with SCI may also require the use of adaptive technologies such as mechanical ventilators or manual or power wheelchairs, all of which greatly affect quality of life (QOL). In addition, individuals with SCI must often cope with an increased incidence of many health problems, such as neurogenic bowel and bladder,^2,3^ respiratory symptoms and complications,^4,5^ cardiovascular complications,^6–8^ pressure ulcers,^9,10^ altered sexual functioning,^11^ urinary tract infections,^10,12^ autonomic dysreflexia,^13–16^ chronic and neuropathic pain,^17,18^ osteoporosis,^19^ and fractures.^20^ Individuals with SCI also often have to cope with altered social roles and psychiatric comorbidities^21–23^ including reactive depression^24,25^ and anxiety disorders.^26^

These issues represent major challenges to living with SCI. The suicide rate for those with SCI is two to six times higher than that of the general population. Further, between 35 and 50 percent of individuals with traumatic SCI have concomitant cognitive difficulties secondary to their injury.^27,28^ Finally, unemployment is also a serious issue in the SCI population, with fewer than 40 % of those under age 65 returning to gainful employment.^29^

Health-related quality of life (HRQOL) refers to the impact of a disease or condition, as well as its associated treatment, on an individual's physical, emotional, and social well-being.^30^ More recent publications have documented the importance of considering the patient's perspective when determining the success of new treatments and interventions.^31,32^ Many of the aforementioned secondary complications and comorbidities are unique to individuals with SCI and significantly impact their HRQOL. However, there is no measure to accurately assess the HRQOL effects of this specific and distinct constellation of medical and mobility issues. The Spinal Cord Injury – Quality of Life (SCI-QOL) measurement system has been developed over the past 7 years to address this unmet need. The goal of the current manuscript is to describe the background and development of the SCI-QOL measurement system.

## Patient reported outcomes and SCI HRQOL research

The need for patient reported outcomes (PROs) assessment is expanding in line with advances in medical treatment that increase life expectancy across chronic and debilitating conditions. Assessments of HRQOL using PROs have become common, if not required, endpoints of many clinical trials, treatment, and intervention programs (e.g. cancer clinical trials).^33^ Unlike clinical outcomes, patient reported outcomes (PRO) assessments measure the impact of health conditions from the patient's perspective. Subjective HRQOL outcomes clearly speak of the patient's needs and expectations.

In spite of these findings, most of the measures used in current SCI research have focused on a single, limited domain (e.g. neurological functioning,^34^ functional independence,^35,36^ participation)^37^ rather than assessing global aspects of HRQOL that may be adversely impacted by a traumatic injury.

Furthermore, SCI researchers have typically used measures of HRQOL developed for the general population.^38–43^ Generic measures often exhibit floor and ceiling effects, contain irrelevant questions that lack validity, and lack the sensitivity needed to detect meaningful differences in the SCI population. For example, commonly administered items on the SF-36 Health Survey^44,45^ ask individuals with SCI about running or climbing several flights of stairs. Similarly, an item on the Satisfaction with Life Scale^46^ asks participants if they were given the opportunity to live their lives over again, would they change almost nothing. These items may offend an individual who has experienced a traumatic injury resulting in permanent, devastating impairments. Such items lack face validity and call into question the utility of generic measures in this population.^26,27^ These measures fail to address unique issues fundamental to the HRQOL of people living with SCI. New methods in measurement development, such as participatory action research,^47–50^ target key stakeholders (in this case, people with SCI) in all phases of measure development and have been tested and validated across other chronic conditions and disabilities, which will certainly improve the content of any forthcoming measures in SCI HRQOL research.

## Traditional SCI outcome measures

Classical outcome measures are also limited by the number of items that can feasibly be included in a measurement scale. Each item takes time to complete, and comprehensive measurement instruments (e.g. WHOQOL-100, the World Health Organization Quality of Life Assessment)^51^ have typically required lengthy administrations, which put an undue burden on the SCI participant with low seating tolerance who is likely experiencing pain and fatigue and may have a decreased attention span. Depending on an individual's neurological level and severity of injury, there is considerable variability in neurologic function following SCI.^52,53^ Because of this extensive range of functioning and capacity, PROs for SCI must therefore have a sufficient number of items to measure HRQOL across a wide range of impairment levels to ensure that the scale is relevant to each individual and has adequate measurement precision at every possible ability level. Thus, there is a tension between the need to develop instruments that are brief and easily administered and the need to develop sufficient items across the full range of each domain.

A scale that contains items measuring too narrow a range of HRQOL is likely to be useful only for a subset of the SCI population. For instance, a PRO scale designed for individuals with high-level tetraplegia is not likely to have sufficient breadth of content coverage when used with individuals who have paraplegia. The content validity of such a scale is questionable when examiners use it across the continuum of impairment seen in the SCI population.

One way investigators have tried to reconcile the need to capture an entire range of HRQOL with respondent burden is to include only a single (or a small number of) item(s) at each distinct point along an ability continuum. In this case, a few items will be very relevant for each respondent, while other items will be less pertinent or not applicable at all to their level of ability in a particular content domain. For example, a person with a neurologically complete C-6 level SCI injury may complete a relevant scale asking about fine motor skills, but will also respond to items about ambulation that are irrelevant. Such irrelevant items yield no useful information, increase time, and sacrifice measurement precision. Modern methods of test development^54^—namely, item banking methodology^55^ combined with computer adaptive testing (CAT)^56^—can help improve these measurement limitations.

## Contemporary methods to improve SCI outcomes measures: using IRT and CAT

The project detailed in this publication, the Spinal Cord Injury Quality of Life measurement system (SCI-QOL), has applied item banking methodology using item response theory (IRT)^57,58^ methods. Such recent advances in PRO measurement science have made it increasingly possible to conduct brief assessments that provide reliable and precise reports of an individual's standing in a given domain (e.g. depression, physical functioning, etc.).

### IRT

Item banking is a prerequisite to using CAT.^54,56^ This multi-stage process consists of selecting or developing a large pool of candidate items, administering the pool of items to a large (e.g. *n* ≥ 200 for a 1-parameter or Rasch model, *n* ≥ 500 for a 2-parameter or graded response model)^59,60^ sample of individuals from the population of interest, and conducting confirmatory factor analyses to confirm unidimensionality of the item pool. Items are evaluated for independence from one another, and items that demonstrate unacceptable (i.e. residual correlation >0.2) local item dependence are removed. IRT analysis^61–63^ is then conducted to flag misfitting items and to calibrate the remaining items (the final calibrated ‘item bank’) based on a single underlying outcome domain. For this project, we utilized graded response model^60^ IRT analysis which estimates, for each item, four location parameters (yielding information on the item difficulty) and a slope parameter (yielding information on the ability of each item to discriminate between participants at different levels of the underlying construct).

IRT, also known as latent trait theory, uses data from individuals with heterogeneous levels of a trait (e.g. depression) to estimate the placement of each item along a single, underlying metric, with less ‘difficult’ items (i.e. those reflecting lower levels of the trait, such as low or nonexistent levels of depression) at one end of this continuum and more difficult (e.g. those reflecting the severe depression) at the opposite end. Calibration involves placing each item into a position on this metric. It is the calibration along this common metric that allows for responses to a subset of items, or even to a single item, to be used to estimate a person's ability level (i.e. amount of the underlying trait) for the entire item bank.

Once calibrated, item banks can be presented through the use of CAT procedures or fixed length short forms that can be customized based on the anticipated level of functioning within a sample, a required level of precision, or a specific participant burden.^54^ The use of item banking procedures combined with CAT delivery represents a major technological advance that has the potential to inform development of an assessment instrument with relevance to all levels of functioning and to increase measurement precision while reducing respondent and administration burden.

### CAT

CAT allows us to estimate scores based on performance of a limited subset of items In practice, this approach minimizes the number of items that need to be administered to an individual to obtain an estimate of functioning. Originally developed in educational and personality testing, CAT methodology uses a computer interface that supplies questions that are tailored to that person's unique ability level. When administered as a CAT, each individual's score for the entire item bank is estimated using a small (e.g. <10) subset of items specifically targeted to their functional level in regard to the underlying trait. For example, a person who is not able to walk 10 feet is not asked to respond to a question about walking 50 feet. An adaptive test asks the most informative question first, generally questions that have high discrimination functions and are in the middle of the ability range. The computer algorithm then selects the next item in the appropriate range of functioning that will have the most discriminating power. With each question the CAT program adjusts its estimate of the person's ability, selecting the questions at the appropriate level of ability or functioning and eliminating unnecessary questions. The program discontinues when either a preset number of items to be administered or a predetermined level of measurement precision is reached, which often requires as few as 4 to 8 items per individual. In summary, CAT employs a simple form of artificial intelligence that selects questions tailored to the test-taker, shortens or lengthens the test to achieve the desired precision, scores everyone on a standard metric so that results can easily be compared, and displays results instantly.

The advantages of applying CAT technology to individuals with SCI are: (1) reduced respondent burden while collecting PRO data in diverse HRQOL areas such as physical, emotional, and social functioning; (2) increased score precision for individuals at all levels of neurological impairment; (3) optimized item selection for each individual; (4) reduction in ceiling and floor effects; (5) person-specific precision estimates across the entire outcome continuum; (6) improved monitoring of data quality in real time; and (7) reduction in data collection costs.

### The development of the SCI-QOL measurement system

Tulsky *et al*. (2011)^48^ and Kisala and Tulsky (2010)^64^ describe a systematic qualitative approach to domain selection and item generation that is centered around individuals with SCI that included individual interviews, focus groups, and cognitive debriefing sessions. The results of this qualitative stage of research were reported earlier.^48^ To ensure linkages with ongoing large scale National Institutes of Health projects and avoid duplication of effort, the team incorporated the item banks that were developed as part of the Neuro-QOL^65^ and PROMIS^66–68^ measurement systems when there was an overlap in constructs (e.g. anxiety, depression), and developed new item banks for domain areas that are targeted to individuals with SCI (e.g. bladder management difficulties) or where there was no suitable PROMIS or Neuro-QOL bank (e.g. resilience).

The SCI-QOL measurement system was developed using IRT^59,69,70^ and includes 19 calibrated item banks and 3 fixed-length scales containing SCI specific items that span the entire range of ability in several HRQOL domains. These features ensure that the instruments have domain relevance and appropriate content coverage. Although individual participants complete only a small subset of the items, their scores are directly comparable to full item bank scores and scores based on any selection of items estimated for a specific trait. Calibrated SCI-QOL item banks can be administered as brief measures that are time-efficient, specific and precise to subgroups, and also flexible with regard to item selection, yet produce total scores that are comparable across a wide range of health and functioning.^54,67^

## Subdomain descriptions

As seen in Table 1, the SCI-QOL is comprised of 22 subdomains across the four broad domains of physical-medical health, emotional health, social participation, and physical functioning. The 22 final subdomains include 19 IRT-calibrated item banks and 3 fixed-length scales. Subdomains were selected and developed based upon qualitative feedback, as well as literature review, and large pools of items were written based on the comments generated by individuals with SCI.^64^ These subdomains then were field-tested, finalized, and programmed into the Assessment Center website (www.assessmentcenter.net).71 All subdomains that are SCI-specific (e.g. Bladder Management Difficulties, Resilience; see Table 1) reference an SCI population. Other, more generic subdomains have been statistically transformed to reference either the Neuro-QOL (e.g. Positive Affect & Well-being) or PROMIS (e.g. Pain Interference) metric and allow for comparison across diagnoses or conditions. Additional detail on the linking procedure may be found in Tulsky *et al*.^72^

**Table 1:** SCI-QOL Banks/Scales: Overview and Availability

SCI-QOL Domains & Item Banks	CAT	Short Form(s)*	Reference Sample	Scoring Direction
Emotional Health				
Positive Affect & Well-being	X	SF10a	Neuro-QOL/General	Better Function
Depression	X	SF10a	PROMIS/General	Severe Symptom
Anxiety	X	SF9a	PROMIS/General	Severe Symptom
Stigma	X	SF10a	Neuro-QOL/Neuro	Severe Symptom
Resilience	X	SF8a	SCI-QOL/SCI	Better Function
Grief/Loss	X	SF9a	SCI-QOL/SCI	Severe Symptom
Self-Esteem	X	SF8a	SCI-QOL/SCI	Better Function
Psychological Trauma	X	SF8a	SCI-QOL/SCI	Severe Symptom
Physical-Medical Health				
Pressure Ulcers *(12-item Scale)*		SF7a	SCI-QOL/SCI	Severe Symptom
Bladder Management Difficulties	X	SF8a	SCI-QOL/SCI	Severe Symptom
Bladder Complications *(5-item Scale)*		*n/a*	SCI-QOL/SCI	Severe Symptom
Bowel Management Difficulties	X	SF9a	SCI-QOL/SCI	Severe Symptom
Pain Interference	X	SF10a	PROMIS/General	Severe Symptom
Pain Behavior *(7-item Scale)*		*n/a*	PROMIS/General	Severe Symptom
Social Participation				
Ability to Participate	X	SF10a	Neuro-QOL/General	Better Function
Satisfaction with Social Roles & Activities	X	SF10a	Neuro-QOL/General	Better Function
Independence	X	SF8a	SCI-QOL/SCI	Better Function
Physical Function (SCI-FI)				
Basic Mobility	X	SF11a	SCI-FI/SCI	Better Function
Ambulation	X	SF11a	SCI-FI/SCI	Better Function
Fine Motor	X	SF9a	SCI-FI/SCI	Better Function
Self-Care	X	SF11a	SCI-FI/SCI	Better Function
Wheelchair Mobility	X	Manual Wheelchair SF10a Power Wheelchair SF9a	SCI-FI/SCI	Better Function

*We have adopted the PROMIS naming conventions for the SCI-QOL short forms The initial form developed for each bank is called the ‘[Name of Bank] SF [# of included items] a’. Subsequent forms that are developed containing different items will be called b, c, etc. unless they are limited to a subset of items in an existing form (e.g., form ‘a’) and would therefore be called ‘[Name of Bank] SF [smaller number of items] a’. For example, the initial Bladder Management Difficulties short form is called ‘SCI-QOL v1.0 Bladder Management Difficulties SF 8a.’ If we developed a shorter form with only 4 of the same items, it would be called ‘SCI-QOL v1.0 Bladder Management Difficulties SF4a.’ If we were to develop an 8-item form containing at least one different item than the form 8a, it would be called ‘SCI-QOL v1.0 Bladder Management Difficulties SF 8b.’ The naming convention applies to ‘official’ short forms Individual investigators/clinicians could develop customized short forms using the associated IRT parameters provided that they have access to psychometric help to develop IRT-based T-score lookup tables for the new form(s).

### Physical-Medical Health subdomains

#### Subdomain 1: Bowel Management Difficulties^73^

This item bank measures a range of difficulties associated with bowel management, including an ability to carry out a bowel program; concerns about incontinence and bowel accidents; concerns about difficulty implementing a bowel program; and the impact of bowel management on everyday living. Toileting issues were cited as having a substantial impact following SCI, and comprised 11 % of focus group comments.^48^

#### Subdomain 2: Bladder Management Difficulties^73^

This item bank measures a range of difficulties associated with bladder management, including ability to carry out a bladder program; worry about bladder accidents; concerns about implementing one's bladder program; and impact on everyday living.

#### Subdomain 3: Bladder Complications^73^

This 5-item fixed-length scale measures a range of difficulties associated with bladder complications including urinary tract infections (UTI) and their impact on spasticity, sexual functioning, and daily living. Originally created as a part of the Bladder Management Difficulties item pool, these items related to UTI comprised a psychometrically distinct factor. These items were calibrated with a reduced sample (*n* = 297) of individuals who had reported a UTI in the past 7 days. Due to the reduced sample size and small number of component items, Bladder Complications was developed as a fixed-length scale rather than as an item bank.

#### Subdomain 4: Pressure ulcers^74^

This fixed-length scale addresses a range of challenges associated with skin care and associated pressure ulcers, such as the extent to which pressure ulcers hinder engagement in social, cognitive, emotional, physical and recreational activities. Skin breakdown following SCI is one of the most significant issues affecting the QOL of individuals with SCI.^48^ The scale consists of 12 test items and one screener and is available in 12- and 7-item versions. These items were calibrated with a reduced sample (*n* = 189) of individuals who reported a pressure ulcer in the past 7 days. Given the small sample size, parameter estimates are likely to be less stable than necessary for implementation of CAT and therefore Skin/Pressure Ulcers was developed as a fixed-length scale rather than as an item bank.

#### Subdomain 5: Pain Interference

This item bank measures the consequences of pain including the extent to which pain hinders engagement with social, cognitive, emotional, physical and recreational activities.^75^ This item bank is comprised primarily of PROMIS Pain Interference items that have been recalibrated for the SCI population. Scores have been transformed to be equivalent to the PROMIS general population referenced metric.

#### Subdomain 6: Pain Behavior

This 7-item fixed-length scale measures manifestations of pain. These actions or reactions can be verbal or non-verbal and involuntary or deliberate. They include observable displays, and verbal reports of pain. This scale includes a small subset of the PROMIS Pain Behavior item bank (*i* = 4) and three new items. These items were calibrated in a SCI sample, but final scores are transformed to the PROMIS general population referenced metric. Due to the small number of component items, Pain Behavior was developed as a fixed-length scale rather than an item bank.

### Emotional Health subdomains

#### Subdomain 7: Depression^76^

This item bank is comprised primarily of PROMIS items, and includes items measuring a feeling of sadness or despair and/or a loss of interest in things as well as feelings of hopelessness, helplessness, and worthlessness. Somatic symptoms (e.g. changes in appetite or sleeping patterns) are not included. This eliminates the possibility that the direct effects that SCI or other secondary medical conditions may have on neurovegetative functioning will spuriously inflate depression scale scores.^77^ Depression and sadness were cited 11 % of the time in focus groups as important factors to one's quality of life.^48^ As with the Pain Interference item bank, the SCI-QOL Depression items were calibrated in a sample of individuals with SCI and final scores were statistically linked^78,79^ to PROMIS depression scores and then underwent a linear transformation to general population PROMIS metric.

#### Subdomain 8: Anxiety^80^

This item bank measures fearfulness, panic, anxious misery, and hyperarousal. General symptoms of anxiety were cited 7 % of the time by focus groups and provide support that the anxiety items in the PROMIS bank are relevant. Additional items were included as participants also mentioned worries and anxiety about engaging in activities that were specific to functioning with SCI.^48^ Like the Pain Interference and Depression item banks, the SCI-QOL Anxiety items were calibrated in a sample with SCI and the final scores were transformed to reflect the general population PROMIS metric.

#### Subdomain 9: Resilience^81^

While traditional emotional factors like depression and anxiety were cited as important constructs that impact QOL, a more frequently cited emotional factor among individuals with SCI was resilience.^48^ Focus group participants used the metaphor of ‘turning the page’ to a new phase of life to describe a critical prerequisite of psychosocial adjustment following injury.^48^ Resilience, the most common response to SCI,^82^ is defined as a subjective experience of adapting to difficult or challenging life experiences, especially highly stressful or traumatic events.^83–85^ SCI-QOL Resilience items address issues such as motivation, coping, and acceptance.

#### Subdomain 10: Positive Affect and Well-being (PAWB)^86^

Positive emotions are universal; all SCI-QOL PAWB items were drawn from the Neuro-QOL item bank of the same name. Neuro-QOL defined PAWB as aspects of a person's life that relate to a sense of well-being, life satisfaction, or an overall sense of purpose and meaning.^65^ In our focus groups, comments related to well-being and life satisfaction were mentioned 9 % of the time, emphasizing the importance of including this construct in a measure of HRQOL. The SCI-QOL item bank was calibrated within our SCI sample then scores were transformed to the Neuro-QOL metric.

#### Subdomain 11: Grief/Loss^87^

This aspect of emotional functioning was prominent in focus group discussions, comprising 14 % of comments, and discussed more frequently than depression or anxiety.^48^ Grief is the natural process of reacting to a loss; the SCI-QOL grief/loss item bank assesses emotional reactions of grief that occur in response to sustaining an SCI, such as anger, guilt, anxiety, sadness, and despair.

#### Subdomain 12: Self-esteem^88^

A common theme of self-esteem was expressed by individuals with SCI, with 11 % of focus group comments focusing on aspects of self-esteem, including self-awareness as individuals compare themselves to an ‘ideal self.’^89^ The SCI-QOL Self-Esteem bank assesses emotional, evaluative, and cognitive perceptions of personal competence and worth. This self-evaluation provides a reference by which to compare oneself to relevant others in social and socially competitive situations.^90^

#### Subdomain 13: Stigma^91^

This is a related construct that was developed as a Neuro-QOL domain. Stigma refers to negative stereotyping that leads to discrimination. It was mentioned in all focus groups, with 3 % of all comments relating to stigma. The SCI-QOL Stigma item bank assesses perceptions of self and publically enacted negativity, prejudice, and discrimination as a result of SCI manifestations. It includes 12 items that were adapted from Neuro-QOL (i.e. with permission, wording has been modified from ‘because of my illness…’ to ‘because of my injury…’). Though the CAT administration order is based on SCI calibrations, the final score has been transformed to the Neuro-QOL metric and in this case reflects a mixed neurological population.^92^

#### Subdomain 14: Psychological Trauma^93^

Psychological trauma results from actual or perceived threat(s) to life, bodily integrity or the mind. It can lead to an overwhelming experience of fear, helplessness or horror, and may render an individual unable to cope effectively. The SCI-QOL Psychological Trauma item bank assesses individuals’ experiences of psychological trauma resulting from SCI, and the symptoms that accompany it.

### Social Participation subdomains

#### Subdomain 15: Ability to Participate in Social Roles and Activities^94^

This domain describes the degree of current involvement in social roles, activities, and responsibilities, including work, family, friends and leisure. The 27-item bank consists exclusively of Neuro-QOL items which were recalibrated with the SCI sample and then transformed to the Neuro-QOL metric.

#### Subdomain 16: Satisfaction with Social Roles and Activities^94^

This domain references satisfaction with involvement in usual social roles, activities, and responsibilities, including work, family, friends and leisure. It contains SCI-targeted items, and items selected from Neuro-QOL and PROMIS (version 1.0). The items were re-calibrated in individuals with SCI and transformed to the Neuro-QOL metric.

#### Subdomain 17: Independence

This refers to perceived independence or ability to communicate one's needs and sense of control over one's life. Seven percent of comments in the Emotional domain focus groups and 5 % of comments in the Social domain focus groups were related to independence and autonomy.^48^ This is an SCI-targeted bank.

### Physical Functioning subdomains^1^ ^95,96^

#### Subdomain 18: Basic Mobility^95,96^

The Basic Mobility item bank contains items about the most basic components of physical functioning. Items in this bank assess individuals’ ability to carry out activities involving changing and maintaining body position, transfers, moving and carrying objects, and moving around in different locations.

#### Subdomain 19: Self-care^95,96^

The Self Care item bank assesses an individual's ability to perform daily self-care activities such as eating, dressing, grooming, and bathing. This bank also contains items assessing the functional components of performing bowel and bladder management programs

#### Subdomain 20: Fine Motor functioning^95,96^

This item bank assesses various components of fine motor functioning including the ability to manually hold, manipulate and move objects that require varying degrees of dexterity and/or strength.

#### Subdomain 21: Wheelchair Mobility^95,96^

This item bank consists of new, wheelchair use-specific items as well as a subset of Neuro-QOL assistive technology items. Items in this bank reflect the ability to transfer in and out of a wheelchair, maneuver a wheelchair under different conditions, engage in activities from a wheelchair and manage wheelchair parts. This bank contains both manual and power wheelchair items, and the CAT version is therefore applicable to users of manual and/or power wheelchairs. In contrast, distinct short forms^97^ are available for manual wheelchair and power wheelchair, respectively.

#### Subdomain 22: Ambulation^95,96^

This item bank is only appropriate for individuals who report the ability to ambulate. Items assess the ability to engage in walking activities in different locations that vary based on speed, time and condition and the ability to manage stairs under different conditions. Some component items specifically reference the use of a walking aid.

### Scoring and administration

IRT-based scores on all SCI-QOL banks/scales use a standardized T metric, with a mean of 50 and a standard deviation of 10. Higher scores on a SCI-QOL item bank represent a greater amount of the construct being measured. For example, for Resilience, a T-score of 60 would represent an individual who is functioning at one standard deviation better than the mean of the SCI population. For Bladder Management Difficulties, a T-score of 60 would indicate an individual performing one standard deviation worse than the mean of the SCI population. The score of 50 represents either the mean of the SCI calibration sample or, for scores transformed to the PROMIS^98^ or Neuro-QOL^92^ metrics, the mean of the US general population.^2^
^2^Note: The SCI-QOL Stigma bank, which is statistically linked to Neuro-QOL, references a mixed neurological population consisting of individuals with stroke, epilepsy, multiple sclerosis, Parkinson's, and ALS.

The use of IRT to calibrate all items in a given bank on a single underlying metric serves to offer a great deal of flexibility in instrument administration. All SCI-QOL CATs are available on the Assessment Center platform and use the same default CAT ‘discontinue’ criteria as PROMIS; namely, the CAT minimum number of items to administer = 4, maximum number of items to administer = 12, maximum standard error = 0.3. Thus, in the default settings, the CAT will always administer at least 4 items, then will discontinue when the standard error of the individual's score estimate drops below 0.3 or a maximum of 12 items is reached, without meeting the standard error variance criterion.

**Figure 1:**
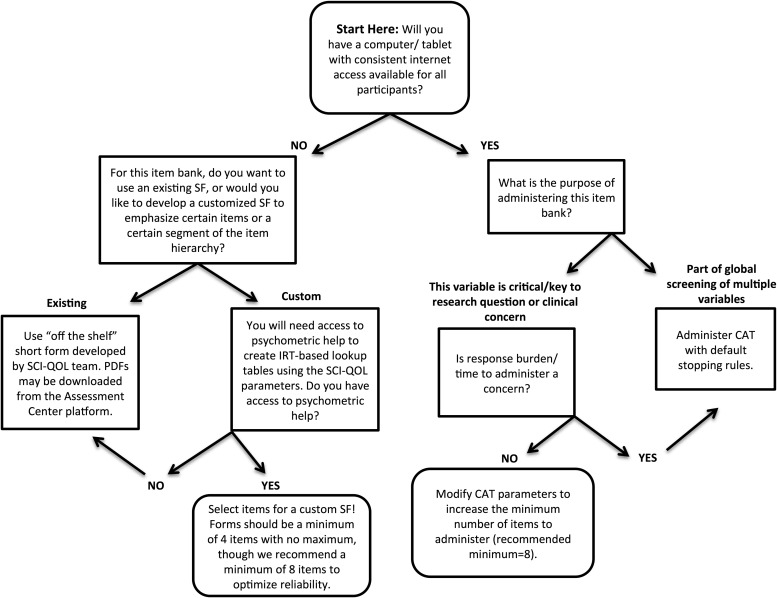
Guidelines for selection of SCI-QOL bank administration method.

Should CAT administration prove impractical in a given clinical or research situation, fixed-length short forms (SF) are available for each item bank. To develop the SFs, we met with co-investigators to review the item information functions produced by the IRT analyses and determined the most discriminating items in the full item bank to include in a short form. We also examined the relative item difficulty (e.g. locations on the measurement continuum) to ensure that we had selected items across the entire continuum of each underlying trait, and balanced these empirical indices with clinical judgment of each item's relative importance. A list of available short forms is available in Table 1. Individual investigators or clinicians can also develop additional, custom short forms, which could then be scored on the same IRT-based metric with the help of a psychometrician. Decision guidelines for selecting between the various methods of administration are shown in Fig. 1. Given the underlying IRT calibrations, standardized SF scores are directly comparable to those obtained via CAT or full-bank administration methods.

### Practical considerations

The SCI-QOL is freely available to the public via the Assessment Center platform or directly from the corresponding author. It will possibly be added to other electronic data capture platforms in the future. A computer or tablet with a consistent internet connection is required to administer SCI-QOL CATs or SFs via Assessment Center. Assessment Center is compatible with the Windows operating system (XP or higher) and with Microsoft Internet Explorer (7 or higher) and Mozilla Firefox (4.0 or higher). An Apple iPad may be used for data collection but not for study administrative functions.^99^ Extensive detail on Assessment Center study setup and administration, including video tutorials, may be found at www.assessmentcenter.net. For researchers or clinicians who are interested in programming the SCI-QOL measures into an alternate CAT administration platform, users should follow all terms of use and copyright restrictions. IRT parameters may be found in the subdomain-specific manuscripts throughout this special issue. Scores are produced for each component item bank/scale (i.e. ‘overall’ or summary scores are not currently available). Finally, the SCI-QOL was developed under the assumption that items and item banks are independent and therefore order of items or measures should not influence scores.

## Discussion

The SCI-QOL measurement initiative was funded with the primary aim of developing a new set of scales of HRQOL that was conceptually grounded in the impact of an SCI on the lives of affected individuals. The SCI-QOL marks the first comprehensive measurement system developed on a large scale that is designed specifically for use in persons with SCI. The SCI-QOL measurement system is comprised of 22 IRT-calibrated banks/scales across physical, emotional, and social functioning. Its design ensures that each item is relevant to individuals with SCI. In some research studies, it may be feasible to administer all 22 item banks/scales; however, this will often not be the case. The importance of any individual bank will depend upon what research question is being asked. We anticipate that the SCI-QOL will be very valuable in clinical settings to identify and detect potential problems and to monitor symptoms. However, further research will help us determine which of the SCI-QOL banks/scales prove most clinically useful.

The SCI-QOL includes targeted items that are specific to individuals with SCI, and also incorporates verbatim PROMIS and Neuro-QOL items so that linkage with these new measurement systems can occur. Each SCI-QOL item bank is constructed to include items across the entire continuum of ability within a HRQOL domain, and can be administered using CAT or SF, making test administration easier and more efficient.

For universally applicable traits already being measured by PROMIS or Neuro-QOL, the SCI-QOL development team carefully reviewed the existing items, developing supplemental, SCI-targeted items as appropriate, empirically tested the relevance of each item in a large SCI sample, developed SCI-specific CAT algorithms to optimize item selection for SCI and, finally, anchored the scores to the relevant PROMIS and Neuro-QOL metric so that a SCI-QOL score would be directly comparable to the PROMIS or Neuro-QOL score for the same domain. Specifically, the SCI-QOL Depression, Anxiety, and Pain Interference item banks are, for all practical purposes, a recalibration of the PROMIS item banks. Similarly, the SCI-QOL Positive Affect and Well-being, Ability to Participate in Social Roles and Activities, Satisfaction with Social Roles and Activities, and Stigma banks are recalibrations of the Neuro-QOL item banks that have been optimized for SCI. ‘Recalibrated’ items have been statistically linked^78,79^ to the PROMIS or Neuro-QOL scores and have then undergone a linear transformation so that SCI-QOL scores on these banks reflect the general population metric. This enhances our ability to compare data across measures for both clinical and research uses.

### Why SCI-QOL?

The development of the SCI-QOL measurement system marks the first time that a comprehensive set of item banks have been developed specifically for use in spinal cord medicine. Typically, measures developed using general population scores contain items that are not relevant while omitting items that are crucial to quality of life in individuals with SCI. Many item banks are unique to individuals with SCI (e.g. bladder management difficulties) and the examiner can only use SCI-QOL as there are no other alternative scales available. For more general banks (e.g. depression, pain interference), there are alternative PROMIS and/or Neuro-QOL item banks that have been developed using general population calibration data. SCI-QOL has optimized these item banks using our SCI-specific sample: irrelevant or poorly performing items have been removed, and CAT algorithms select items based on data from our SCI sample. For this reason, we highly recommend administering the SCI-QOL version of each of these banks.

### Clinical and research applications

In a clinical or research context, SCI professionals can document the initial impact (e.g. grief/loss and other psychological response) of SCI; in the less acute phase, researchers and clinicians can use the SCI-QOL to document changes and trajectories of recovery. Finally, in the longer term, viable intervention and rehabilitation targets can be established. In the future, the SCI-QOL measures may have policy-level implications, in terms of helping to derive the financial and HRQOL impact of SCI and its associated disability.

### Study limitations and future directions

The SCI-QOL has focused development on 22 conceptual subdomains that are relevant to individuals with SCI. These subdomains were selected from qualitative data obtained from individuals with SCI. Other banks could have been developed, but this work fell beyond the scope of this project. The use of qualitative methods of domain/subdomain selection will only be as good as what our focus group discussed at the meetings. Some potentially important subdomain areas (e.g. cognitive functioning) may be too difficult to discuss in a group setting.^27,28^

The papers that follow in this special issue provide the initial evidence of the reliability and internal consistency of the SCI-QOL (e.g. IRT-based internal consistency and test-retest reliability)^72^ as well as its initial validation. All of the development work followed a rigorous item development and evaluation process. We have ensured the content validity of each SCI-QOL item bank by involving ‘experts’ – individuals with SCI and SCI clinicians – at each step throughout the development process. All items are relevant to individuals with SCI and we evaluated the item wording and content with formal cognitive testing/debriefing procedures. The items in the final bank are all interrelated and seem to be measuring the same construct. When tested with CFA, the items fit a unidimensional model. Moreover, each of the papers in this issue provide results that all items fit a 2-parameter IRT model. Any item showing poor item fit or DIF was removed. The stability coefficients from the IRT model are high as are test-retest correlation coefficients obtained in a separate sample that completed the measure twice.

At the same time, the data provided in this issue provide only the initial validation and psychometric evidence. Construct validation of any test involves the process of marshaling evidence from several sources and over time.^100^ We recommend new studies that evaluate the relation between SCI-QOL scales and existing instruments (e.g., convergent and discriminant validity of each of the SCI-QOL banks) as well as studies of the SCI-QOL's responsiveness to change. Future work should include the development of a global/summary SCI-QOL score. Most important, the SCI-QOL instruments have enormous potential to serve as clinical instruments and the development of indices that have clinical meaningful should be a high priority. For instance, studies that determine clinical ‘cut points’, minimally important clinical differences, and indices of reliable clinical change are needed. Furthermore, once these clinical indices are developed, SCI-QOL scores could be incorporated into electronic medical records so that they could be used in clinical practice.

## Conclusion

The remaining papers in this special issue provide extensive detail on the development and testing of each item bank/scale as a primary source for information on each SCI-QOL item bank/scale. The following papers provide all the technical details of the development process. The papers also provide practical details that will facilitate use of SCI-QOL in research and clinical practice.

## Disclaimer statements

**Contributors** All listed authors participated in the design/conduct of this study and in the preparation of the content of this manuscript.

**Funding** This work was co-funded by the National Institute of Child Health and Human Development/National Center on Medical Rehabilitation Research and the National Institute of Neurological Disorders and Stroke (NINDS) (Grant #5R01HD0054659). Additionally, funding for the development of the SCIFI physical functioning item banks was provided by the National Institute on Disability and Rehabilitation Research (Grant #s H133N060022, H133N060024, H133N060014, H133N060005, H133N060027, and H133N060032), and funding to expand the calibration sample into the VA was provided by the Department of Veterans Affairs, Rehabilitation Research & Development National Center of Excellence for the Medical Consequences of Spinal Cord Injury (Grant #B8212-C).

**Conflicts of interest** Dr Tulsky owns a copyright to the tools being used in this study. The purpose of the copyright is to protect the integrity of the tool. Currently, the item banks are available for free and there are no immediate plans for him to benefit financially from the copyright.

**Ethics approval** Institutional IRB approval was received at each participating site.

## Footnotes

1 The Spinal Cord Injury – Functional Index (SCI-FI) is an SCI-specific set of 5 IRT-calibrated item banks measuring physical functioning that were developed with funding from the National Institute on Disability and Rehabilitation Research as part of a collaborative Model SCI Systems project. Four new SCI-FI/AT item banks are described in this issue.
